# Environmental Aspects of Domestic Cat Care and Management: Implications for Cat Welfare

**DOI:** 10.1155/2016/6296315

**Published:** 2016-09-28

**Authors:** Judith L. Stella, Candace C. Croney

**Affiliations:** Center for Animal Welfare Science, Purdue University, West Lafayette, IN, USA

## Abstract

Domestic cats (*Felis silvestris catus*) are the most commonly kept companion animals in the US with large populations of owned (86 million), free-roaming (70 million), research (13,000), and shelter (2-3 million) cats. Vast numbers of cats are maintained in homes and other facilities each year and are reliant on humans for all of their care. Understanding cat behavior and providing the highest quality environments possible, including positive human-cat interactions, based on research could help improve the outcomes of biomedical research, shelter adoptions, and veterinary care, as well as overall cat welfare. Often, however, cats' needs are inadequately met in homes and some aspects may also not be well met in research colonies and shelters, despite the fact that similar problems are likely to be encountered in all of these environments. This paper provides a brief overview of common welfare challenges associated with indoor housing of domestic cats. Essential considerations for cage confinement are reviewed, along with implications of poor cat coping, such as weakening of the human-animal bond and relinquishment to shelters. The important role that environmental management plays in cat behavior and welfare outcomes is explored along with the need for additional research in key areas.

## 1. Introduction: Factors Contributing to the Welfare Problem of Cat Relinquishment

Recent statistics from the American Society for the Prevention of Cruelty to Animals (ASPCA) and the Humane Society of the United States (HSUS) estimate that there are approximately 86 million owned cats and between 50 and 70 million feral and/or free-roaming cats in the United States [1, 2]. Two to three million of these enter shelters each year, of which 70–75% are euthanized.

Through a series of studies by The National Council on Pet Population Study and Policy [3] and others, several reasons for relinquishment and return of cats have been identified. These typically include abandonment/stray (31%), owner circumstances (move, illness, divorce, and financial) (19%), unwanted kittens (14%), and allergies (5%) [4]. Behavioral reasons are thought to be another leading cause of cat relinquishment (approximately 12%) [5, 6], with the most common of these including house soiling, problems between pets, aggression toward people, unfriendliness, fearfulness, and destructive behavior. The owners level of knowledge about species-typical cat behavior also appears to be a factor in relinquishment. Fewer of those who relinquished cats knew that they pounce, scratch, or bite as a form of play and that the number of cats in the home affects cat behavior; relinquishers also exhibited knowledge deficits about cat estrous cycles [7]. Weak owner attachment, not having owned another cat as an adult, and having unrealistic expectations of a particular role for the cat to fill also contributed to surrendering [5]. Additionally, cats at higher risk of relinquishment were younger, mixed breed, and sexually intact. Cats kept confined to basements or garages most of the day, those maintained without access to the outdoors, and those primarily cared for by an adult woman had an increased risk for relinquishment [5].

Interestingly, the welfare of cats in homes is not usually addressed to the extent that it is within a research colony or a shelter, although similar problems are likely to arise in all environments in which cats are confined. For example, Morgan and Houpt [8] found the most common behavior problems of cats in US homes to be scratching furniture (60%), eating houseplants (42%), conspecific aggression (36%), food stealing (25%), hissing/aggression to people (17%), house soiling (16%), excessive vocalizations (16%), fabric chewing (7%), and “shyness” (4%) [8]. Similarly, Heidenberger's [9] investigation of cats in homes indicated that the most frequent behavior problems cited by cat owners, via a questionnaire, were anxiety (16.7%), scratching furniture (15.2%), feeding problems (10.9%), aggression (10.5%), inappropriate urination (8.2%), and defecation in the house (5.1%) [9]. The most frequently mentioned anxiety-inducing stimulus was a visit by strangers. Neutered females, cats adopted between the ages of 5–12 months, and cats weighing greater than 4 kg were more likely to be anxious. Cats acquired from a shelter, as a stray, or from a friend all tended to show anxiety more often than those born in the owner's home. The number of cats in the home and the amount of available space per cat also were relevant, with multiple cat households and limited space per cat both leading to homes more likely to have a cat described as anxious. Many of these behavioral issues have been observed in response to inappropriate environments under laboratory conditions as well [10].

International studies of cat behavior report similar findings. Amat et al.'s [11] study of 336 cats referred to a behavior clinic in Spain noted that problem behaviors presented for treatment were similar to those cited as problems in the home or as reasons for relinquishment [11]. These included aggression (47%), inappropriate elimination (39%), compulsive behavior (3.5%), excessive vocalization (2.5%), fear and phobias (2.5%), and “other,” which was comprised of anorexia, scratching furniture, and overactivity (5.4%). In agreement with other studies, intercat aggression (64% of aggression cases) was found to be a common problem. This may be a species-typical behavioral response for a solitary animal that finds itself unable to disperse and also lacks a well-developed intraspecific communication system, as proposed by Leyhausen and Lorentz [12]. However, many common behavior problems, particularly aggression and inappropriate elimination, are not tolerated well by owners.

Collectively, these studies suggest that owner attention to meeting cats' needs may be the most important determinant of welfare outcomes in homes, given that owner decision-making dictates all of the cat's living conditions. While it is possible that cat behavioral problems may simply be normal behaviors that are unwanted by owners, it is also likely that exhibition of “problem” behaviors could be in response to a poor quality environment or one in which the cat is unable to cope. According to Turner [13] “many behavioral problems result from a lack of consideration of the needs of the cat, poor or changing housing conditions, unrealistic expectations of the owner or inadequate interactions between the owner and the cat” [13]. Regardless of the reason, inadequate housing and handling diminish welfare for the cat.

However, owner self-reports suggest that many do not adequately provide for their cats' needs. For example, Heidenberger [9] found that 24% of companion cats in homes did not have their own food bowls and over half had to share the litter pan with other cats, both of which may lead to resource guarding and defensive behavior in cats. Not surprisingly, cats in groups of two or three exhibited more problem behaviors than did single cats. Having outdoor access was negatively associated with problem behavior, with owners reporting few to no problems with cats that could go outside. Additionally, the quality of owner-cat relationships is often highly variable, despite the finding that those who interact often and regularly with their cats on a daily basis report fewer problem behaviors [9].

Taken together, these findings highlight the need for greater owner attention to cats' behaviors and their overall needs. In particular, understanding the role of the environment in cat behavior and its relationship to the human-animal bond and owner satisfaction is essential to facilitate positive cat-human interactions. Such information may improve retention in homes and better support the welfare of cats in all environments in which they are maintained. The aim of this paper is to outline environmental considerations for confined cats that will lead to improved cat welfare.

## 2. Macroenvironmental Considerations

Various aspects of the environment may affect the welfare of the cat when confined in homes or cages in shelters, veterinary hospitals, or research facilities. Those of particular importance to cats include the physical components of the macro- and microenvironments and the social environment, which includes the quality of human-animal interactions.

The macroenvironment refers to the cat's housing space (room, building, or barn) and its surroundings and includes factors such as the thermoregulatory environment, lighting, odors, and sounds [14]. Although the thermoregulatory environment exerts a major influence on animal welfare, cats may be unable to express temperature regulating behaviors because of a lack of resources available to them to do so and often the thermoneutral zone of the species is not adequately considered in their housing. For example, the thermoneutral zone for domestic cats is 30–38°C [15] (NRC 2006). Yet most cat housing areas in homes and laboratories are maintained closer to 22 ± 2°C [15]. Thus, thermal discomfort may be a common experience for many cats, despite being an issue that is relatively easy to remedy. Providing opportunities for cats to behaviorally thermoregulate such as provision of warm bedding, resting areas, boxes, or heating elements such as SnuggleSafe® will enable them to more easily cope with the environment.

Another macroenvironmental factor that impacts cat well-being in various housing environments is odor. Because almost all mammals depend more on olfactory cues than do humans, aversive odors can be a source of chronic stress for confined animals. For cats, potentially objectionable odors include the scent of dogs (natural predators of cats), unfamiliar conspecifics, alcohol, cleaning chemicals (including laundry detergent), and citrus scents [16].

Another factor to consider is sound frequency range and intensity. The auditory frequency range of cats exceeds that of humans [17], making assessment of the welfare implications of high frequency noise difficult. Sound intensity in savannah and rain forest habitats ranges from 20 to 40 dB [14], whereas it regularly exceeds 100 dB in shelters and laboratories during routine husbandry [18]. Furthermore, sound intensity of 73 dB has been found to activate the stress response system of rats, leading to a 100–200% increase in plasma corticosterone levels [19]. Based on these findings, it is likely that reducing noise levels and maintaining sound intensity around 60 dB (quiet conversational level) may be beneficial to cats.

## 3. Microenvironmental Considerations

The micro or cage environment must also be considered relative to animal welfare. Microenvironmental factors include usable floor space, food presentation, elimination facilities, and outlets for the expression of species-typical behaviors. The type, presentation, and availability of these features of the environment can be a source of either stress or enrichment to cats [14, 16].

A particularly important microenvironmental factor to consider is the quantity and quality of space provided to cats. Confined cat spaces or housing environments are often reduced in both quantity and quality of space in comparison to options available to their wild or free-roaming counterparts. Although recommendations for minimum cat cage sizes have been published [20, 21], their basis is questionable as scientific evidence of the welfare implications (particularly adverse consequences) of keeping cats in smaller than recommended cages is not readily available. Thus, it is possible that cats may actually need larger or smaller minimum space allocations than what are currently recommended. Further to this point, the need to provide cats more than the recommended minimum of 0.56 square meters of floor space (6 square feet) is often discussed in animal sheltering communities. However, few studies have been conducted that might help establish precise minimum requirements for short- and long-term cage confinement of cats and thus provide a more informed basis for recommended space allocations for cats.

Recent studies have indicated that the quality of the environment may be more relevant to the cat than the size of the cage during both short and long periods of cage confinement [10, 22–24]. Further, Stella's [24] investigation of the behavior of cats housed in cages providing 1.1 square meters (11.8 square feet) of floor space found no difference in the number of sickness behaviors or in time to adaptation in the first 48 hours than cats housed in cages half that size [23]. Thus, while a minimum cage size clearly exists that affords cats reasonably good welfare, more work is needed to determine optimal cage sizes that also accommodate furnishings which permit both freedom of movement and the ability of cats to engage in species-typical behaviors for which they are highly motivated. In the interim, cats housed in cages for longer periods of time in shelters or research facilities may benefit from being provided daily exercise periods outside of their cages.

Whether kept in homes or other facilities, the type of shelter offered to cats should permit partial isolation from conspecifics and people, as this may be of critical importance to some, enabling them to feel a sense of security that would otherwise not occur [16]. Additionally, variation in the height at which cats can navigate their home environments also appears to be an important component of cat housing. Cats seem to prefer to monitor their surroundings from elevated vantage points and usually welcome provision of climbing frames, hammocks, platforms, raised walkways, shelves, or window seats [25]. Additional furnishing of the environment is often necessary to promote cat health and well-being. Appealing, appropriate objects must be provided to confined cats to permit expression of behaviors that include scratching and marking, which maintain claw health, and to leave both visual and pheromonal territorial marks [16, 25]. In short, the captive environment should be behaviorally relevant, with the quantity and quality of space provided allowing for the development and normal expression of species-typical behavioral patterns.

Another key element is the availability, type, and presentation of food offered to cats. For most cats under human care, food is typically provided in the form of a formulated, uniform, and consistent diet and placed in a single location so that the animal's time and energy related to foraging behavior are greatly reduced. Consequently, boredom that manifests as over- [26] or undereating [10, 23] may result. Attending to how cats are fed therefore becomes an important component of their behavioral and overall welfare management, whether kept singly or in groups and regardless of the type of housing environment in which they find themselves. Offering food in interactive puzzle feeders can provide mental and physical enrichment and is one strategy that may be implemented to minimize boredom and promote exercise.

## 4. Cat-Human Interactions

As noted previously, the quality and quantity of human-animal interactions experienced by cats are both relevant to their welfare outcomes in various settings. In captivity, acclimation to human presence is an important fitness-determining factor since humans select for tameness and other behaviorally acceptable traits; individuals that do not meet such criteria are often prevented from reproducing. A human-animal relationship can be said to exist if a number of repeated interactions between the animal and human occur, eventually allowing each to make predictions about the other's behavior. Both positive and negative human-animal relationships are important in the context of animal welfare, and this concept is as applicable to cats as to other species. In human-animal relationships, the human generally dictates the number and nature of interactions and hence the relationship, while the animal more often simply reacts to the human's actions.

The predominant reaction of many animals when exposed to humans is fear, and it has been proposed that this occurs because animals often perceive encounters with humans as predatory [27]. Fearful responses can lead to negative caretaker attitudes toward their charges, increasing the likelihood of poor interactions recurring. Given that people's attitudes towards cats are often ambivalent and that several openly express some dislike of cats [28], the caretaker-cat dynamic may be especially vulnerable and in need of consideration. An animal's fear of people can be reduced and desirable behaviors increased, even after receiving poor treatment, especially with attention to offering consistent positive human-cat interactions. These include utilizing low-stress handling techniques or feeding preferred food items [29].

As with all other aspects of confinement, control and predictability of caretaker behaviors are of great importance to the animal's perceptions of humans [30]. Likewise, in effective cat management, a familiar person appears to be essential. Wild felids are considered to be sensitive to the captive environment, which can result in large numbers of abnormal behaviors and decreased reproductive success. This can be ameliorated by improved keeper-cat relationships. Mellen [31] noted a positive correlation between the quality of keeper interactions and increased reproductive success in small captive felids [32]. Wielebnowski et al. [33] found a negative correlation between fecal cortisol concentrations and the amount of time the primary keeper spent with clouded leopards and a positive correlation between fecal cortisol and the number of keepers. The interpretation of these results was that a higher number of keepers prevented the animals from forming and maintaining predictable relationships with any of the keepers, thus increasing the stress of captivity [33]. As a result, consistent, positive human-animal interactions may facilitate improved cat welfare.

Finally, the social environment is of great importance to cats. The social behavior of domestic cats exhibits great plasticity. It appears to be influenced by ontogeny such that kittens socialized to other cats, humans, dogs, and so forth during the sensitive period of socialization are likely to adapt to life in social groups more readily than are kittens raised by their mothers alone [34]. This social plasticity appears to be distributed across the family Felidae as illustrated by a study of 16 species of small Felidae from five lineages which found that the expression of affiliative behavior toward humans was widely distributed, rather than concentrated in the domestic cat lineage [35].

## 5. Welfare of Cats Confined in Cages

Each year millions of cats are housed in cages in veterinary hospitals, shelters, and research laboratories. Therefore, understanding aspects of the cage environment that facilitate or prohibit the ability of cats to cope may potentially impact the welfare of large numbers of individuals. Novelty, confinement and the inability to express species-typical behaviors may result in cats experiencing distress [32]. Their related responses may include decreased appetite, withdrawal from social groupings, increases in salivary, blood, and fecal cortisol, increases in urinary cortisol: creatinine ratios, decreases in grooming, and increases in the frequency and intensity of attempts to hide [10, 22, 36]. Medical interventions (e.g., vaccinations, treatment for parasites, and neutering), while potentially beneficial to the cat's physical health, can introduce additional stressors and thus impact the psychological health of the cat.

Because cats evolved in environments where hiding was an adaptive response to threat of predation, it is likely that “pet” cats also display such behavior in threatening environments, like veterinary hospitals and shelters. Because thwarting attempts to hide may contribute disproportionately to overall causes and related measures of stress [37], one form of environmental enrichment often suggested to help cats to cope with confinement has been provision of hiding and perching opportunities. For example, McCune [38] and Rochlitz [25] demonstrated that the ability to hide may be essential to cats when exposed to stressors [25, 38]. Hiding behavior, which is correlated with enhanced ACTH response and increased urinary cortisol concentrations, has been identified as a key indicator of cat stress [36]. These studies suggest that not allowing cats the opportunity to hide may adversely affect their welfare.

In shelters, the idea that allowing cats to hide decreases their chances of adoption often overrides this welfare concern. One study [39] aimed to determine if adding a hide box improved cats' abilities to cope with the environment, allowing the cat to become more comfortable and interactive with unfamiliar people. It was found that cats that were provided boxes approached more often and retreated less than did control cats (those with no box). They were also more often seen sleeping restfully than controls. In contrast, control cats exhibited more vigilance behavior, which is problematic as vigilance has been associated with anxiety-related behavior problems in house cats. Cats in the enriched group were observed in or on their hide boxes 77% of the time, whereas control cats attempted to hide 36% of the time. There was no difference in time to adoption between the groups, disproving the rationale for not providing cats with a hide box. Importantly, cats appeared to be coping, indicated by lower Cat Stress Scores, by day three, whereas control cats exhibited behaviors indicative of a change to chronic stress by the end of the two-week study period. Vinke et al. [40] likewise demonstrated the importance of affording cats the opportunity to hide as means of coping with environmental stressors [40]. Shelter cats provided with a Hide Perch and Go® box were found to acclimate more quickly to a new environment compared to those without a hiding area based on their Cat Stress Scores, suggesting that it provided an effective form of enrichment that facilitated coping [40].

It should be noted that the Cat Stress Score (CSS) is a tool that is often used to assess stress in cats, which describes seven possible stress levels based on cat body postures and behaviors (see [41] for details). Despite its frequent use, it has been proposed that what is really being measured is fear, as evidenced by the three highest scores being labeled as “fearful,” “very fearful,” and “terrorized.” Additionally, it incorrectly assumes that there is a reliable and accurate way to “measure” stress in cats [42]. Therefore, this caveat should be considered when interpreting the results of studies utilizing such scoring.

Nonetheless, given the limited tools available for evaluating stress in cats, CSS was used in another study [43] examining the responses of cats in four different treatments: single housing with usual care, single housing with enrichment, communal housing with usual care, and communal housing with enrichment. Results indicated that CSS were similar in all groups on day one, but thereafter cats in single housing with usual care had higher CSS than all other groups. They also had the lowest adoption rates and the longest length of time waiting for adoption, and they exhibited more fearful behavior than did cats in the other groups. In this study both housing and handling were manipulated, so either could have produced the effect seen.

In addition, Ottway and Hawkins [44] tested the hypothesis that cats in long-term shelter care housed in groups of unfamiliar conspecifics experience diminished welfare (higher CSS) due to unstable and inappropriate social groupings. A comparison of 12 adult cats unfamiliar with each other, communally housed in a large run, and cats that were either singly housed or pair housed with a familiar conspecific (former housemate) was conducted. The results indicated that the CSS was higher in cats housed communally than in cats housed in single units or with previously familiar conspecifics. Communally housed cats spent more time hiding than single housed cats (26% versus 4%). Play behavior was only observed in 1% of the observation periods and exclusively in singly housed cats or in cats housed with familiar conspecifics. It was therefore concluded that cats housed communally experienced higher levels of stress than cats housed in discrete units and they had more difficulty coping, probably due to the instability of the group, with unstable groups being more stressful than group living itself [44]. Similarly, de Monte and le Pape [45] concluded that for adult cats single housing may not be considered a “totally unfortunate housing situation,” especially if the cats have daily positive interactions with humans [45].

The domestic cat has often been used as a model for those interested in identifying and addressing welfare problems in wild felids because these exotic species are easily distressed in captivity. Carlstead et al. [36] imposed a 21-day psychological stressor on singly housed domestic cats that included unpredictable caretaking and mildly aversive handling, a chronic psychological stressor for confined cats. Stressed cats exhibited decreased activity levels and increased attempts to hide compared to controls. They also had increased adrenocortical output (increased urine cortisol concentrations), enhanced adrenal sensitivity to ACTH, and reduced pituitary sensitivity to luteinizing hormone-releasing hormone. The researchers concluded that the environment led to activation of the stress response system in the cats and that hiding was an important behavior for modulating HPA axis activation caused by an unpredictable environment [36].

Similarly, McCobb et al. [46] evaluated stress levels among cats in usual and enriched housing via behavioral assessment (CSS) and monitoring of urine cortisol: creatinine ratios in four different shelters. Results indicated that cats housed in enriched environments had lower stress levels than those housed in traditional shelters. Stress levels among the cats were highest in the morning and decreased throughout the day. A slight negative correlation between the number of days spent in the shelter and the CSS was found with the CSS decreasing with increasing time spent in the shelter. In agreement, the mean morning CSS of the cats in the holding areas was higher than that of the cats in the adoption area. No differences were found between the CSS of owner surrenders and strays. Additionally, 24% of the cats had signs of systemic disease including upper respiratory infections, vomiting, and diarrhea. While no significant relationship was found between the noise level at the shelter and CSS, cats that were housed where they could see, hear, and/or smell dogs had higher urine cortisol: creatinine ratios. Additionally, almost 25% of cats had signs of systemic illness and more than 25% of the urine samples collected had trace amounts of hematuria. The authors concluded that the biggest factor affecting the cats' stress levels in the different types of shelters appeared to be the extent to which they were exposed to dogs. Cats in areas with more exposure to dogs had higher CSS than did cats in other high noise areas. Exposure to dogs appeared to have a cumulative effect on cat health when combined with other environmental stressors in that it increased stress levels more in cats that were obviously ill than in those that had no signs of disease [46]. Stella et al. [23] similarly observed distress in cats in noisy rooms with exposure to disturbances that included recorded sounds of dogs barking [23]. These findings provide strong evidence of the need for both enrichment and consistent management of the cat's environment, particularly with regard to noise to avoid causing cats undue distress and consequently adverse health conditions.

Rochlitz et al. [47] assessed the quarantine experience of cats over six months and observed that the cats required two to five weeks to acclimate to the quarantine situation. The authors concluded that hiding was an important behavior expressed by cats confronted with an aversive situation, such as a novel environment [47]. The withdrawal of friendly human contact was particularly distressful to cats used to receiving a lot of attention and may be important in shelter environments as well and potentially may be worse for owner surrender cats than for strays. Dybdall et al. [48] subsequently investigated this in a study designed to assess the social history of the cats admitted to the shelter. The CSS was used, and the observers were blinded to which group (owner surrender or stray) the cat belonged. Cats were scored for the first three days of housing while in the holding area. No effect of gender or neuter status was found. However, cats surrendered by their owners had higher CSS than did stray cats. Overall, cats that were deemed suitable for adoption had lower CSS than did cats that were deemed unsuitable and subsequently euthanized. Moreover, cats in the owner surrender group became ill significantly sooner than cats in the stray group did [48]. In agreement with the Rochlitz et al. [47] findings, this study indicated that all cats experienced a stress response associated with entry to the shelter, but the owner surrender cats may have experienced an additional psychosocial stressor of forced social separation from their primary caretakers and home environments. Alternatively, owner surrender cats may come from an unfavorable environment that led to behavior problems and relinquishment and may already be more distressed than strays at the time of admission.

Finally, Kessler and Turner [41] assessed cat acclimation to boarding over two weeks and compared the boarding cats' CSS to those of control cats living in a shelter. They evaluated single, pair, and group housing situations. The results indicated that two-thirds of the cats acclimated, one-third found boarding distressful, and 4% never acclimated. Thus, boarding was deemed inappropriate for that group. The daily CSS of the singly housed cats declined significantly from day one to day five, and overall stress levels continued to decrease during the two weeks of boarding [41]. However, in agreement with the findings of earlier studies they never reached the level of the control cats. This is an important finding since cats in shelters may not have time to acclimate before being rehomed. In fact, most failed adoptions and returns take place within two weeks of adoption. The period of greatest risk for cats appears to fall within the time they are acclimating to the new environment, indicating that current protocols may not be sufficient to allow cats to fully adjust to the new environment and thus impact cat welfare.

## 6. Conclusions

In summary, it has been suggested that cats do not meet all the criteria for domestication and may best be described as “exploited captives” [49]. Confinement of cats, in homes or other environments, may lead to poor welfare through inadequate environments that do not meet the needs of cats. Ultimately, the environmental needs of the cat are similar whether they are confined to a home or a cage in a shelter, research facility, veterinary hospital, or boarding facility. Aspects of the environment that can be perceived as potential threats or aversive stimuli whether parts of the macro- or microenvironment, human-animal interactions, the social environment, or the predictability and control of the environment all interact to influence a cat's well-being. Poor welfare may be reflected in poor physical health, illness, and disease or behavioral problems such as house soiling and fearful and aggressive behaviors. These factors may lead to a breakdown in the human-animal bond and ultimately to abandonment, relinquishment to a shelter, or euthanasia and thus require further investigation. Research is needed to refine recommendations for the quantity of space needed by confined cats kept both singly and in groups and to better understand the interactions between quantity and quality of space provided to cats. Simple enhancements to improve the quality of cats' living quarters via enrichment such as hiding areas may yield many beneficial effects. Studies on the short- and long-term effects of improving the quality of the housing environment and human-cat interactions on adoption rates, retention outcomes, and even infectious disease incidence are needed to improve cat well-being. In addition, research focused on identifying and understanding the effects of individual differences in coping styles could lead to further improvements in cat welfare. Finally, the etiology and role of owners' attitudes and knowledge about cats are needed to reduce risks to the human-animal bond and to optimize cat well-being.
